# Electroacupuncture Attenuates Neuropathic Pain and Comorbid Negative Behavior: The Involvement of the Dopamine System in the Amygdala

**DOI:** 10.3389/fnins.2021.657507

**Published:** 2021-05-07

**Authors:** Xue-Hui Zhang, Chen-Chen Feng, Li-Jian Pei, Ya-Nan Zhang, Liu Chen, Xu-Qiang Wei, Jia Zhou, Yue Yong, Ke Wang

**Affiliations:** ^1^Acupuncture Anesthesia Clinical Research Institute, Yueyang Hospital of Integrated Traditional Chinese and Western Medicine, Shanghai University of Traditional Chinese Medicine, Shanghai, China; ^2^Department of Anesthesiology and Research Institute for Acupuncture Anesthesia, Shuguang Hospital Affiliated to Shanghai University of Traditional Chinese Medicine, Shanghai, China

**Keywords:** electroacupuncture, neuropathic pain, negative emotion, dopamine system, amygdala

## Abstract

Neuropathic pain (NeuP) is an important clinical problem accompanying negative mood symptoms. Neuroinflammation in the amygdala is critically involved in NeuP, and the dopamine (DA) system acts as an important endogenous anti-inflammatory pathway. Electroacupuncture (EA) can improve the clinical outcomes in NeuP, but the underlying mechanisms have not been fully elucidated. This study was designed to assess the effectiveness of EA on pain and pain-related depressive-like and anxiety-like behaviors and explore the role of the DA system in the effects of EA. Male Sprague-Dawley rats were subjected to the chronic constrictive injury (CCI) model to induce NeuP. EA treatment was carried out for 30 min once every other day for 3 weeks. The results showed that CCI caused mechanical hyperalgesia and depressive and anxiety-like behaviors in rats and neuroinflammation in the amygdala, such as an increased protein level of TNFα and IL-1β and activation of astrocytes. EA treatment significantly improved mechanical allodynia and the emotional dysfunction induced by CCI. The effects of EA were accompanied by markedly decreased expression of TNFα, IL-1β, and glial fibrillary acid protein (GFAP) in the amygdala. Moreover, EA treatment reversed CCI-induced down-regulation of DA concentration, tyrosine hydroxylase (TH) expression, and DRD1 and DRD2 receptors. These results suggest that EA-ameliorated NeuP may possibly be associated with the DA system to inhibit the neuroinflammation in the amygdala.

## Introduction

Neuropathic pain (NeuP) is a disabling condition that is induced by peripheral and/or central nerve injury and is estimated to affect between 100 and 560 million people worldwide (Baron, 2006; Alles and Smith, 2018). NeuP is a major health concern that can significantly reduce quality of life and pose a huge clinical burden, and it has become a major public health problem throughout the world (Toth et al., 2009). Like all chronic pain, NeuP is characterized not only by persistent and intractable pain but is also associated with negative emotions, such as anxiety and depression (Khan et al., 2020). The reciprocal facilitative interactions between pain sensitivity and negative emotions are much more common than previously thought, with a 30–60% co-occurrence rate (Maletic and Raison, 2009). Because of the coexisting physical and mental conditions, people with chronic pain have a higher rate of suicidal tendencies than the general population (Kirtley et al., 2020). A simultaneous intervention on pain-modulating pathways and mood/affect networks is considered to be an effective treatment for NeuP (Mitsi et al., 2015). One meta-analysis of clinical trial data even strongly recommended that tricyclic antidepressants and serotonin-noradrenaline reuptake inhibitor antidepressants should be used as the first-line therapy for NeuP (Finnerup et al., 2015). However, clinical treatment is still challenging because of limited efficacy and many side effects of pharmacological therapy, and NeuP therefore can be refractory. Ultimately, many patients seek help from complementary and alternative medicine treatments, such as acupuncture.

Electroacupuncture (EA) is used worldwide and provides clinically significant benefits in patients with various types of pain, including NeuP (Zhang et al., 2014; Vickers et al., 2018). Recently, a systematic review concluded that EA achieve better efficacy in treating chronic pain with depression than medicine therapy (Yan et al., 2020). A 3 years follow-up study showed that EA combined with a low dose of fluoxetine hydrochloride was beneficial in treating chronic persistent somatoform pain (Bai et al., 2017). Previous preclinical studies have shown that EA not only effectively alleviates pain but also significantly improves pain-related depression- and anxiety-like behaviors in animal models of chronic pain (Li et al., 2014; Xu et al., 2020). However, the underlying mechanisms of EA in treating pain and accompanying negative emotions remain unclear.

The amygdaloid complex (also known as the amygdala) is an important part of the limbic system because it is critical in regulating emotion and motivation (Janak and Tye, 2015). Electrophysiological studies in animals and functional imaging studies in humans have shown increased responsiveness of the amygdala during physiological pain episodes (Neugebauer, 2015). The amygdala displays adaptive changes in activity and neuroplasticity during chronic pain, which subsequently affect pain modulation and the emotional-affective dimension of pain, and facilitates neuropathic pain processing (Rouwette et al., 2012). Lesions or inhibition of special amygdala neurons alleviated mechanical allodynia and pain affective-motivational behaviors induced in NeuP models (Arimura et al., 2019; Corder et al., 2019; Ji and Neugebauer, 2020). It is well known that excessive neuroinflammation is one of the key mechanisms for generating and sustaining chronic pain (Ji et al., 2014). Accumulating evidence suggests that high levels of neuroinflammation occur in the amygdala (Burke et al., 2013; Guan et al., 2020). Recently, emerging evidence has suggested that the dopaminergic system plays a critical role in regulating motivational and emotional behavior, including mood and pain modulation (Yang et al., 2020). Animal and human studies confirmed that the dopaminergic system could regulate the excitability of amygdala neurons (Kroner et al., 2005; Bergman et al., 2014). Moreover, dopamine (DA) is not only an important neurotransmitter but also an important regulator of immune and inflammatory responses. For example, DA activates dopamine D1 receptor (DRD1) to alleviate neuroinflammatory injury by inhibiting the NLRP3 inflammasome (Yan et al., 2015). The activation of DRD2 also suppresses neuroinflammation through αB-crystalline in the central nervous system (Shao et al., 2013).

The amygdala is an important direct-response region involved in EA effects (Fang et al., 2009; Wang et al., 2016), and the endogenous dopaminergic system can be regulated by EA (Torres-Rosas et al., 2014; Lin et al., 2019). Previous studies, including our own, have confirmed that EA can reduce neuroinflammation in animal models of neuropathic pain (Liang et al., 2016; Wang et al., 2018). Therefore, we aimed to explore whether EA alleviation of allodynia and negative behavioral states was associated with the modulation of the dopaminergic system in inhibiting neuroinflammation in the amygdala in a rat model of chronic constrictive injury (CCI).

## Materials and Methods

### Animals

Male Sprague-Dawley (SD) rats (240–260 g) were purchased from Shanghai Slack Laboratory Animal Ltd. (Shanghai, China). The animals were housed in a controlled environment with temperature at 21 ± 1°C and relative humidity of 60–70% under a 12-h light/dark cycle with food and water available *ad libitum*. The rats were allowed to acclimatize to their new surroundings for 1 week before experiments. The 39 rats were randomly divided into three groups: (1) sham group: all operations were performed without CCI; (2) CCI group: CCI-induced NeuP model; and (3) EA group: CCI-induced NeuP model with EA stimulation. All procedures followed the National Institutes of Health Guidelines for the Use of Laboratory Animals and were approved by the Institutional Animal Care Committee of Yueyang Hospital of Integrated Traditional Chinese and Western Medicine, Shanghai University of Traditional Chinese Medicine, Shanghai, China (YYLAC-2019-047).

### CCI Model

The chronic constriction injury of the sciatic nerve procedure was performed on the left side in the rats according to the method described in our previous study (Ju et al., 2013). Rats were anesthetized via intraperitoneal injection with sodium pentobarbital (40 mg/kg). The left sciatic nerve was exposed at the mid-thigh level and tied four times by gut suture (4-0 silk). In the sham group, we exposed only the left sciatic nerve without ligature. All surgical procedures were performed by the same experimenter in order to avoid any possible bias with regard to operator variability. There was no mortality in this CCI model.

### EA Treatment

Stainless steel needles were inserted at a depth of 6 mm into bilateral ST36 and GB34 acupoints. The ST36 acupoint is located 2 mm lateral to the anterior tubercle of the tibia, and the GB34 acupoint is located in the depression anterior and inferior to the fibula capitulum. The two ipsilateral needles were connected to the output terminals of the HANS Acupuncture Point Nerve Stimulator (LH-200; Beijing Huawei Industrial Developing Company, Beijing, China). The frequency was set at 2 Hz, and the intensity of stimulation was increased stepwise from 0.5 to 1.0 and then 1.5 mA, with each step lasting 10 min. Each EA stimulation lasted 30 min in total. On the basis of our own previous study using the same model, the maximum allodynia was apparent 7 days after surgery (Ju et al., 2013). Therefore, EA treatment was applied at day 8 after CCI. The rats received EA stimulation once every other day for 8–28 days (total of 11 times).

### Behavioral Tests

#### Nociceptive Behavioral Test

Mechanical withdrawal thresholds (MWT) were assessed using an electronic von Frey plantar aesthesiometer (IITC, Woodland Hills, CA, United States). Baseline values were obtained before CCI surgery. The rats were placed individually in Plexiglas cages with a gridded floor for 30 min before the test to ensure acclimation. As described previously, the central part of the left hindpaw was stimulated, and paw withdrawal, flinching, or licking was considered to be a positive behavior (He et al., 2019). Three stimuli were applied at the same time point at intervals of 3 min. Each value was recorded, and MWT were represented by the mean values.

#### Open Field Test

The open field test (OFT) is used to assess locomotor activity and anxiety-like behavior (Prut and Belzung, 2003). Rats were brought into the behavior assessment room and were allowed to habituate there for 30 min. The testing apparatus was a transparent (100 cm length × 100 cm width × 40 cm height) box with a non-reflective black floor. Each rat was gently placed in the central zone and allowed to freely explore the area for 30 min. Time, entries, and distance traveled in the central zone (defined as 60% of the total area of the box) and the total distance traveled during the test were recorded using a digital video camera and measured using SMART 3.0 software (Panlab, Cornella, Spain). Between tests of individual rats, the apparatus was cleaned using 75% ethanol.

#### Elevated Plus Maze Test

The elevated plus maze test (EPMT) is conducted to measure anxiety induced by open spaces and height (Linnerbauer et al., 2020). The maze consisted of four 50 × 10 cm arms connected by a 10 × 10 cm common center area. Two opposite-facing arms were open, whereas the other two opposing arms were enclosed by 40 cm high walls. The maze was placed 80 cm above the floor in a test room. Each rat was placed onto the center area with the head toward one open arm and allowed to explore the maze for 5 min. Time and distance in the open arms and head dips in open arms were recorded and analyzed with the SMART software. After testing, the apparatus was cleaned as described above.

#### Forced Swimming Test

In the forced swimming test (FST), a glass cylinder (30 cm height × 18 cm diameter) was filled with 23 ± 1°C water. Individual rats were placed into the cylinder for 6 min, and immobility behavior in the last 4 min test session was recorded. Immobility was defined as the rat’s floating without struggling and making only those movements necessary to keep its head above water (Colonna and Butovsky, 2017).

The experimenter was blinded to both the injury condition of the animal and the EA treatment in all behavioral trials.

### Determination of DA, TNFα, and IL-1β Concentration With Enzyme-Linked Immunosorbent Assay

All animals were sacrificed. DA levels were detected in the amygdala using an enzyme-linked immunosorbent assay (ELISA) kit (Eagle Biosciences, Nashua, NH, United States) according to the manufacturer’s instructions. The levels of TNFα and IL-1β in the amygdala were also determined with ELISA kits (Shanghai Yuanye Biotechnology, Shanghai, China).

### Western Blotting

The amygdala samples were harvested 24 h after final behavioral test. The amygdala of rats was ultrasonically disrupted in a cold radio immunoprecipitation assay (RIPA) lysis buffer (Beyotime Biotechnology Co., Haimen, Jiangsu, China) with protease inhibitors (PMSF, Beyotime Biotechnology Co., Haimen, Jiangsu, China) followed by centrifugation at 12,000 × *g* for 20 min. Then, the total protein concentration of the supernatant was quantified by a BCA kit (Beyotime Biotechnology Co., Haimen, Jiangsu, China). Western blotting was performed as described previously (Hiroi et al., 1997). Primary antibodies used were tyrosine hydroxylase (TH, rabbit polyclonal, 1:2,500; Proteintech, Chicago, IL, United States); glial fibrillary acid protein (GFAP, rabbit polyclonal, 1:3,000; Abcam, MA, United States); Iba1 (rabbit monoclonal, 1:1,000; Abcam); DRD1 (rabbit monoclonal, 1:2,000; Abcam); DRD2 (rabbit polyclonal, 1:1,000; Abcam, MA, United States); and glyceraldehyde-3-phosphate dehydrogenase (GAPDH) (rabbit monoclonal, 1:5,000; Abcam, MA, United States). Secondary antibodies were horseradish peroxidase-conjugated goat anti-rabbit (1:5,000; Abcam, MA, United States) and horseradish peroxidase-conjugated goat anti-mouse (1:5,000; Abcam, MA, United States).

### Immunohistochemistry and Immunofluorescence

Briefly, the PFA-fixed brain tissues containing the amygdala were dissected on a rodent brain matrix (ASI Instruments, Warren, MI, United States), paraffin embedded, and sectioned at 5-μm thickness using a sliding microtome (Leica Microsystems, GmbH, Wetzlar, Germany). Every fifth section was used for immunostaining for DRD1 (rabbit polyclonal, Abcam), DRD2 (mouse monoclonal, Santa Cruz Biotechnologies), or GFAP (mouse monoclonal, Servicebio Technology Co., Ltd.). Dewaxed sections on the slides were incubated in 0.01 M phosphate-buffered saline (PBS) containing 3% H_2_O_2_ and 40% methanol for 30 min at room temperature and then treated with 0.5% Triton X-100 (Sigma Chemical Co.) in PBS for 5 min. The sections were washed with PBST (0.01 M PBS with 0.0.1% Triton X-100) three times and treated with 1% BSA and 0.05% Triton X-100 for 2 h at room temperature. For immunohistochemistry, the sections were incubated with a primary antibody directed against GFAP (1:500) overnight at 4°C, followed by an incubation with biotinylated goat anti-mouse IgG (Vector Laboratories Inc.; 1:200) for 2 h at room temperature. The immunohistochemically stained sections were transferred to an avidin–biotin–peroxidase complex (Vector Laboratories Inc., 1:200) for 2 h and then developed in 0.05% 3,3′-diaminobenzidine (DAB) (Sigma Chemical Co.) and 0.003% H_2_O_2_ in PBS. Then, sections were dehydrated in ethanol, cleared in xylene, and coverslipped with Neutral balsam. The images were recorded using an Olympus microscope (BX 61) equipped with a DP70 digital camera. For immunofluorescence, the sections were incubated with a primary antibody directed against DRD1 (1:1,000) or DRD2 (1:300) overnight at 4°C, followed by an incubation with fluorescent-labeled goat anti-rabbit or goat anti-mouse IgG (1:600, Servicebio Technology Co., Ltd.). After three washes in PBST, the immunofluorescence-stained sections were coverslipped and photographed. These signaling were quantified using ImageJ software application (1.46r version).

### Statistical Analyses

Statistical analyses were performed with analysis software SPSS 19.0 (IBM Corp., Armonk, NY, United States). All results are expressed as the mean ± standard error (SEM). Differences in MWT data among the groups at various time points were compared by two-way repeated-measures analysis of variance with the Bonferroni *post hoc* test for pairwise multiple comparisons. The OFT, EPMT, FST, ELISA, western blotting, immunohistochemistry, and immunofluorescence data were subjected to one-way analysis of variance (ANOVA) followed by the Newman–Keuls or Tukey *post hoc* test. A *p* < 0.05 was considered statistically significant.

## Results

### EA Ameliorates Mechanical Allodynia Induced in CCI-Induced NeuP

**FIGURE 1:**
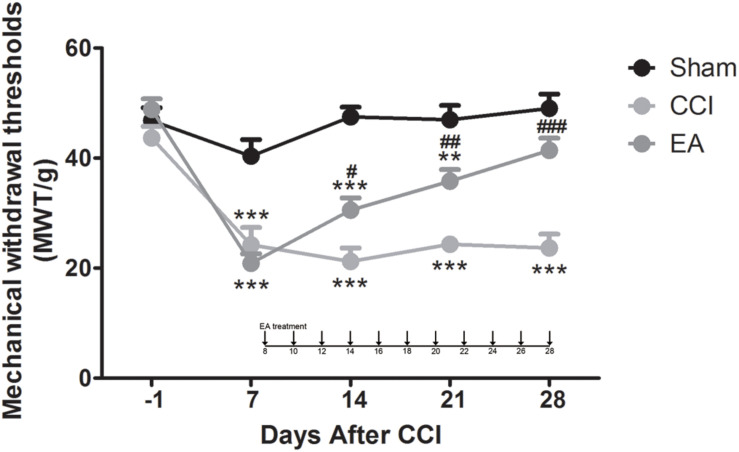
The effects of electroacupuncture (EA) on mechanical allodynia induced by chronic constrictive injury (CCI) in Sprague-Dawley (SD) rats. EA treatment was applied at day 8 after CCI. The rats received EA stimulation once every other day for 8–28 days (total of 11 times). Evaluations of mechanical withdrawal thresholds in the sham group, CCI-induced neuropathic pain (NeuP) model group (CCI group), and CCI-induced NeuP model with EA treatment group (EA group). All data are expressed as the mean ± SEM (*n* = 13 per group). **p < 0.01, ***p < 0.001 vs. the sham group; ^#^*p* < 0.05, ^##^*p* < 0.01, ^###^*p* < 0.001 vs. the CCI group.

For mechanical allodynia, a two-way repeated-measures ANOVA revealed a significant main effect of treatment [*F*(2, 144) = 69.42, *p* < 0.0001] and a significant group × time interaction [*F*(8, 144) = 7.263, *p* < 0.0001]. On day -1, before the CCI surgery, there were no significant differences in MWT among the three groups (*p* > 0.05). After the rats received the CCI surgery (post-CCI), the MWT of their ipsilateral hindpaws decreased significantly on day 7 compared with the sham group (all *p* < 0.001; Figure 1), which indicated that CCI induced a prominent mechanical allodynia. EA stimulation significantly increased the MWT from day 14 to day 28 (overall *p* < 0.05, EA versus CCI; Figure 1). These data indicated that EA alleviated the mechanical allodynia in the rat CCI model.

### EA Improved Emotional Disorders Caused by CCI

**FIGURE 2:**
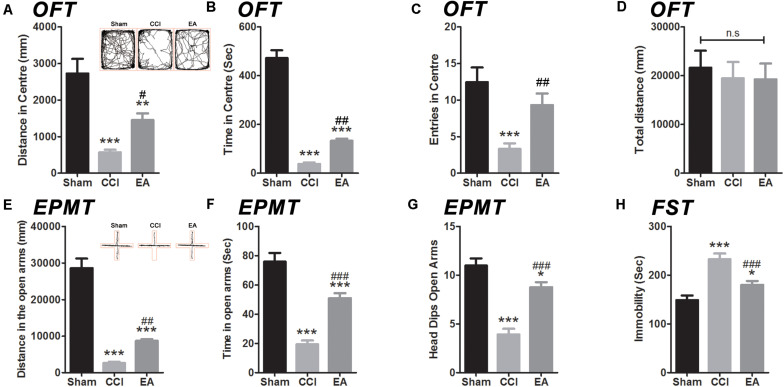
The effects of EA on depressive and anxiety-like behaviors induced by CCI rats. The anxiety-like behavior was accessed by both in the open field test (OFT) **(A–D)** and elevated plus maze test (EPMT) **(E,F)**. **(A)** Distance in center of the OFT; **(B)** time spent in center of the OFT; **(C)** entries in center of the OFT; **(D)** total distance in the OFT; **(E)** distance in the open arms of the EPMT; **(F)** time spent in the open arms of the EPMT; **(G)** the frequency number of head dips (EPMT). The depressive-like behavior was in the forced swimming test (FST) **(H)**. All data are expressed as the mean ± SEM (*n* = 13 per group). *p < 0.05, **p < 0.01, ***p < 0.001 vs. the sham group; ^#^*p* < 0.05, ^##^*p* < 0.01, ^###^*p* < 0.001 vs. the CCI group. The “n.s” means no statistical differences were observed among three groups.

In order to ascertain if EA treatment has an effect in negative behavioral states induced by CCI, we performed several behavioral tests from day 29 to day 31 using the OFT, EPMT, and FST, respectively (Figure 2). In the OFT, the rats in the CCI and EA groups traveled shorter distances [*F*(2, 36) = 69.42, all *p* < 0.01], spent shorter time [*F*(2, 36) = 135.9, all *p* < 0.001], and had fewer center entries [*F*(2, 36) = 9.029, all *p* < 0.01] in the central zone than the sham group (Figures 2A–C). Total distance did not differ in all groups [*F*(2, 36) = 0.1505, all *p* < 0.01] (Figure 2D). After 3 weeks of EA treatment, rats traveled longer distances (*p* < 0.05) and spent more time (*p* < 0.01) in the central zone than those in the CCI group (Figures 2A,B). In the EPMT, the rats in the CCI and EA groups also traveled shorter distances [*F*(2, 36) = 79.74, all *p* < 0.001], spent less time [*F*(2, 36) = 45.08, all *P* < 0.001], and had fewer entries [*F*(2, 36) = 34.18, all *p* < 0.05] in the open arms than the rats in the sham group (Figures 2E–G). Compared with the CCI group, rats treated with EA traveled longer distances (*p* < 0.01), spent more time (*p* < 0.001), and had a lower frequency of head dips (*p* < 0.001) in the open arms (Figures 2E–G). Regarding the FST, the immobility time was increased in the CCI group [*F*(2, 36) = 19.59, *p* < 0.001], which was also higher than that in the EA group (*p* < 0.001) (Figure 2H). Together, these data suggested that EA treatment could attenuate CCI-induced depressive and anxiety-like behaviors.

### EA Alleviated the Inflammatory Response in the Amygdala

**FIGURE 3:**
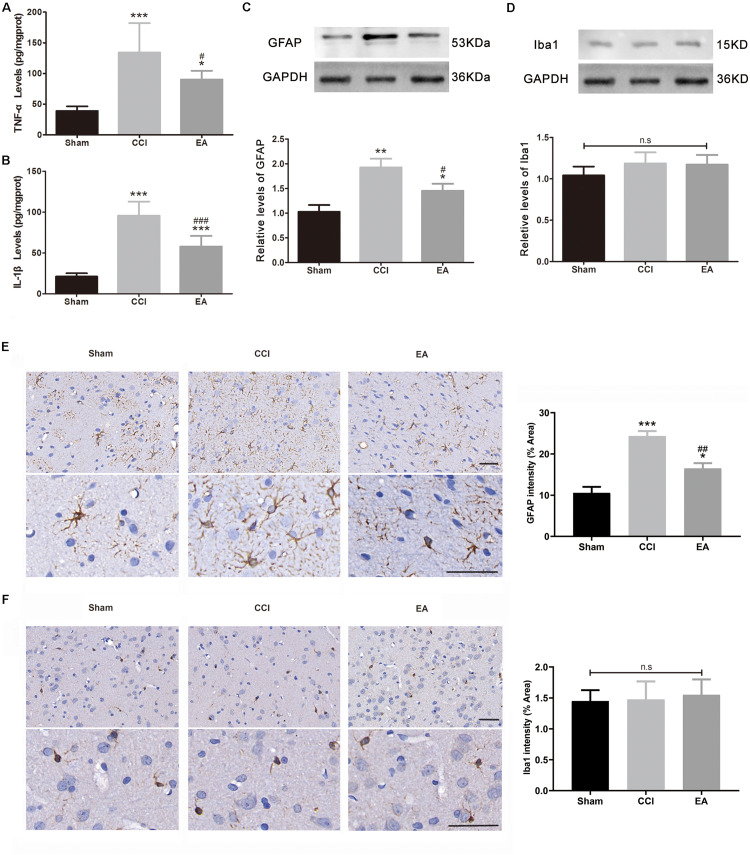
The effects of EA on neuroinflammation in the amygdala. The concentration of TNFα **(A)** and IL-1β **(B)** was analyzed using enzyme-linked immunosorbent assay (ELISA) (*n* = 6 per group). The protein levels of glial fibrillary acid protein (GFAP, **C**) and Iba-1 **(D)** were analyzed using western blotting, while the **(C,D)** used the same loading control (GAPDH) (*n* = 3 per group). Representative immunohistochemistry staining images and signal intensity quantitation of GFAP **(E)** and Iba-1 **(F)** are shown (scale bar = 50 μm) (*n* = 4 per group). Results are expressed as the mean ± SEM. *p < 0.05, **p < 0.01, ***p < 0.001 vs. the sham group; ^#^*p* < 0.05, ^##^*p* < 0.01, ^###^*p* < 0.001 vs. the CCI group. The “n.s” means no statistical differences were observed among three groups.

The levels of TNFα [*F*(2, 15) = 15.74, *p* < 0.001] and IL-1β [*F*(2, 15) = 52.04, *p* < 0.001] in the amygdala were significantly increased in the CCI group compared with the sham group (Figure 3). EA treatment significantly reduced the production of TNFα (*p* < 0.05) and IL-1β (*p* < 0.001) (Figure 3). Astrocytes and microglia are the key regulators of neuroinflammation and are increased/activated during neuroinflammation to secrete inflammatory cytokines (Colonna and Butovsky, 2017; Linnerbauer et al., 2020). GFAP and Iba1 are specific biomarkers for astrocytes and microglia, respectively. As shown in Figures 3C,E, GFAP levels in the amygdala were significantly increased in the CCI group compared with the sham group [*F*(2, 6) = 25.92, *p* < 0.001, western blot; *F*(2, 9) = 26.62, *p* < 0.001, immunohistochemistry staining], while the EA treatment significantly reduced the level of GFAP (*p* < 0.05). The Iba1 protein expression showed no significant difference between groups [*F*(2, 6) = 1.336, *p* = 0.3311, western blot; *F*(2, 9) = 0.0454, *p* = 0.9558, immunohistochemistry staining; Figures 3D,F].

### EA Increased DA and Regulated the DRDs

**FIGURE 4:**
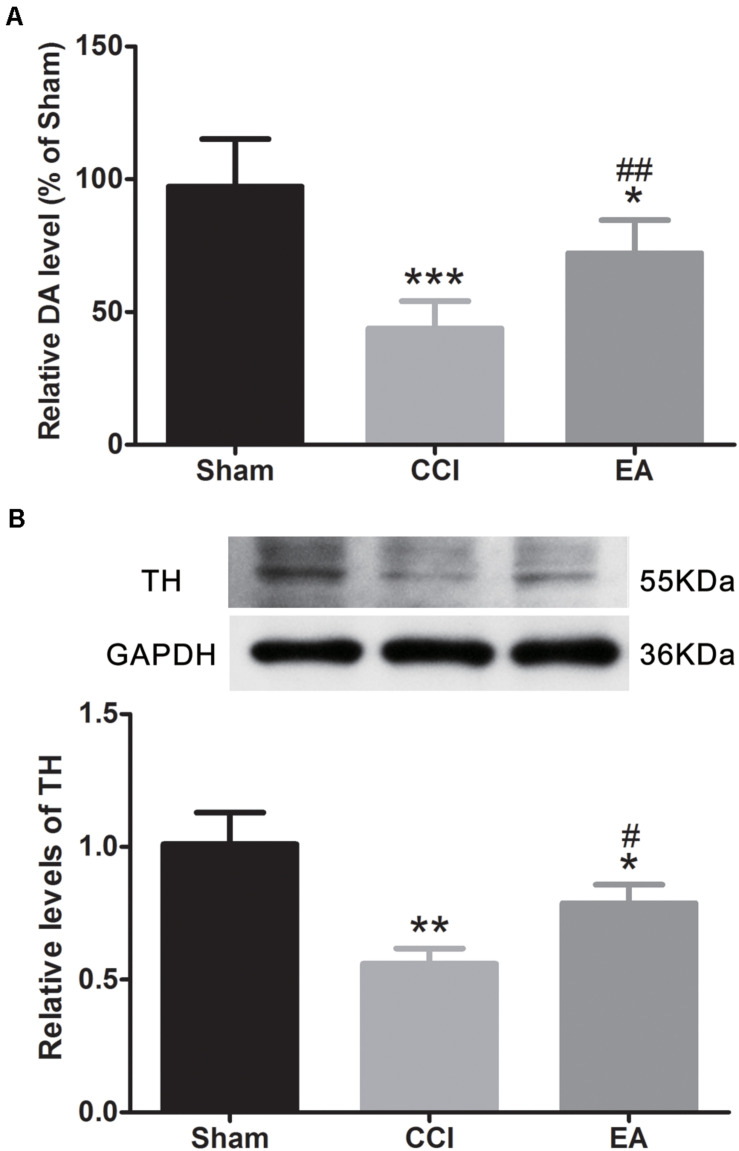
The effects of EA on dopamine (DA) concentration and tyrosine hydroxylase (TH) protein expression in the amygdala. **(A)** Quantitative analysis of DA concentration (*n* = 6 per group); **(B)** western blot images and quantification showing the protein level of TH (*n* = 3 per group). All values are the mean ± SEM. *p < 0.05, **p < 0.01, ***p < 0.001 vs. the sham group; ^#^*p* < 0.05, ^##^*p* < 0.01 vs. the CCI group.

**FIGURE 5:**
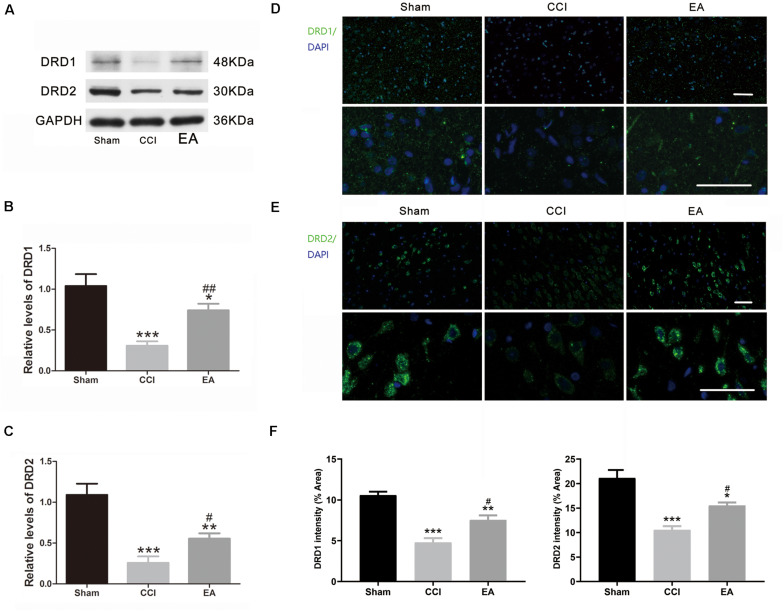
The effects of EA on DRD1 and DRD2 protein expression in the amygdala. **(A)** Western blot gel images showing protein levels of DRD1 and DRD2 in the amygdala. Quantification of DRD1 **(B)** and DRD2 **(C)** in the amygdala, respectively (*n* = 3 per group). Representative immunofluorescence staining images of DRD1 **(D)** and DRD2 **(E)** in the amygdala sections and signal intensity quantitation **(F)** are shown (scale bar = 50 μm) (*n* = 4 per group). Data are presented as the mean ± SEM. *p < 0.05, **p < 0.01, ***p < 0.001 vs. the sham group; ^#^*p* < 0.05, ^##^*p* < 0.01 vs. the CCI group.

Compared with the sham group, the DA concentration in the amygdala was significantly decreased in the CCI group [*F*(2, 15) = 22.21, *p* < 0.001], which was significantly reversed by EA treatment (*p* < 0.01) (Figure 4A). Since TH is the rate-limiting enzyme for the biosynthesis of DA (Hiroi et al., 1997), we examined the expression of TH in the amygdala. As shown in Figure 4B, the expression of TH in the CCI group was significantly lower than that in the sham group [*F*(2, 6) = 20.16, *p* < 0.01], while EA significantly elevated the expression of TH (*p* < 0.05) (Figure 4B). The available evidence suggests that DRD1/DRD2 signaling is involved in neuroinflammatory processes (Yan et al., 2015; Qiu et al., 2016). Therefore, we evaluated whether changes in the expression of DRD1 and DRD2 in the amygdala also were affected by EA treatment. As shown in Figures 5A–C, the DRD1 and DRD2 protein levels were significantly decreased in the CCI group as compared to the sham group [DRD1 expression: *F*(2, 6) = 40.07, *p* < 0.001; DRD2 expression: *F*(2, 6) = 53.94, *p* < 0.001]. EA treatment also significantly increased DRD1 (*p* < 0.01) and DRD2 (*p* < 0.05) protein levels as compared to the CCI group. Immunofluorescence staining of DRD1 and DRD2 on the brain section further verified that the expression of DRD1 and DRD2 increased in the amygdala [DRD1 expression: *F*(2, 9) = 20.30, *p* < 0.001, Figures 5D,F; DRD2 expression: *F*(2, 9) = 18.36, *p* < 0.001, Figures 5E,F].

## Discussion

The present study demonstrated that EA treatment alleviated nociceptive and behavioral impairment associated with CCI-induced NeuP. Meanwhile, EA inhibited CCI-induced neuroinflammation in the amygdala. In addition, EA reversed the decreased concentration of DA and the expression of TH and restored the reduction of DRD1 and DRD2 in the amygdala induced by CCI. These results suggest the effects of EA on the sensory and behavioral impact of NeuP might be associated with the modulation of DA system in the amygdala.

NeuP is usually accompanied by hyperalgesia and allodynia and could potentially trigger anxiety and depression as a result of long-term pain in both patients and animal models (Descalzi et al., 2017; Khan et al., 2020). The present study showed that CCI induced a mechanical allodynia and obvious anxiety- and depressive-like behaviors in OFT, EPMT, and FST tests. Our results showed that EA treatment relieved the mechanical hyperalgesia and anxiety- and depressive-like behaviors. The results are consistent with the previous studies showing that EA has analgesic, antianxiety, and antidepressant-like effects on NeuP (Li et al., 2014; Jang et al., 2020).

Clinical observations and experimental studies demonstrate that the effect of acupuncture is closely related to regulate the nervous system, including peripheral, spinal, and supraspinal levels (Zhao, 2008; Zhang et al., 2014). Previous studies showed that the spinoparabrachial tract and the spinothalamic tract are the two main pathways that mediate and integrate acupuncture signals from the periphery to the supraspinal level (Zhao, 2008). The spinoparabrachial tract originates from the superficial dorsal horn in the spinal cord and projects to the parabrachial nucleus connecting to other brain areas. The spinothalamic tract originates in the superficial and deep dorsal horn and projects to the thalamus connecting to the cortical areas. Furthermore, recent studies demonstrated that EA stimulation at hindlimb regions can drive the activation of the sciatic nerve, vagal efferents, and spinal sympathetic pathway (Torres-Rosas et al., 2014; Liu et al., 2020). The amygdala receive the neural projection from the thalamus and parabrachial nucleus and also have neural connections between each other (Thompson and Neugebauer, 2017). This may be a potential mechanism by which EA stimulation at hindlimb regions can affect the amygdala.

It has been well-documented that the amygdala plays a critical role in on-off switching in NeuP (Rouwette et al., 2012). Evidence is increasing that activating immune and inflammatory processes has an important role in the induction and maintenance of chronic pain along with increasing pain and negative effect (Ji et al., 2014; Walker et al., 2014). Proinflammatory cytokines TNFα and IL-1β could alter the neural activity of the amygdala, which is implicated in the pathophysiology of pain, depression, and anxiety (Ming et al., 2013; Prossin et al., 2015; Klaus et al., 2016). Our results showing that exposure to CCI increased TNFα and IL-1β in the amygdala is consistent with these findings. One study showed that suppressing inflammatory cytokines in the amygdala could alleviate the anxiety-like behavior in a chronic pain mouse model (Guan et al., 2020). The administration of IL-1β-neutralizing antibody prevents the spared nerve injury-induced memory deficits and depressive and pain behaviors (Gui et al., 2016). Meanwhile, local infusion of TNFα-neutralizing antibody in the amygdala reverses anxiety-like behavior in mice with persistent inflammatory pain (Chen et al., 2013). Additionally, EA or transcutaneous auricular vagus nerve stimulation (taVNS) treatment was found to improve depressive-like behavior and reduce pain intensity in CCI rats and was also associated with inhibiting the expression of TNFα in the amygdala, prefrontal cortex (PFC), hippocampus, and hypothalamus (Guo et al., 2020). Our results showed that EA significantly inhibited the up-regulation of TNFα and IL-1β in the amygdala. Overactive astrocytes and microglia cells are the major contributors to neuroinflammation, synthesizing and releasing TNFα and IL-1β (Clarkson et al., 2017). In our present study, we showed that CCI induced the dramatic activation of astrocytes in the amygdala, while microglia were unaffected. The activation of astrocytes usually promotes the activation of microglia, and there is a synergistic effect between the two glial cells (Jha et al., 2019). A study on spatiotemporal activation of miroglia in CCI-associated affective disorder model showed that microglia are not generally activated in the whole brain (Barcelon et al., 2019). This study found that microglia will not be activated at 1 and 4 weeks after CCI in the amygdala. At delayed time points, which is 8 weeks after CCI, microglia are indeed activated in the amygdala, while we only detected the activation of microglia at 4 weeks post CCI modeling because the behavioral anomalies have showed up then. The microglia may also be activated at a later point, and the astrocyte activation may be the initiator. In this study, EA treatment could alleviate the astrocyte activation in the amygdala.

DA is a crucial anti-inflammatory mediator in peripheral and central inflammation (Xia et al., 2019). EA could elevate circulating DA levels to control systemic inflammation (Torres-Rosas et al., 2014). Recent research has also indicated that the brain DA system is an important immediate effector in EA treatment (Ye et al., 2017; Lin et al., 2019). Additionally, the amygdala has dopaminergic innervation and DA receptors, and DA is released in the amygdala in response to both physiological and pathophysiological stimuli (Lee et al., 2017). Our research showed that EA elevated the concentration of DA and the expression of TH in the amygdala, suggesting that DA is likely involved in the effect of EA. DRD1 and DRD2 are major pharmacological targets for neuroinflammation. For instance, DA inhibits nucleotide-binding oligomerization domain-like receptor pyrin domain-containing 3 (NLRP3) inflammasome-dependent neuroinflammation by binding to DRD1/cyclic adenosine monophosphate (cAMP) signaling (Yan et al., 2015). The activation of DRD2 has well-established anti-neuroinflammatory effects through a αB-crystallin-dependent mechanism (Shao et al., 2013). Our results showed that the inhibition of CCI-induced neuroinflammation of the amygdala by EA was associated with an increase in the DRD1 and DRD2 levels, consistent with the trend in DA expression level. Immunofluorescence staining of DRD1 or DRD2 with the nuclear dye DAPI on the brain section showed that the increased expression of DRD1 and DRD2 was mainly probably in the neural fibers and pericaryon, respectively. The existing evidence shows that the DRD1 is mainly distributed in basolateral (BLA) amygdala and the posteroventral region of the medial amygdala (MeApv), and the DRD2 is mainly expressed in the central nuclei (CeA) of the amygdala (Kim et al., 2018; Miller et al., 2019). Immunohistochemical staining showed that the DRD1 and DRD2 are expressed in astrocytes (73%) rather than neurons (19%) (Pavlova et al., 2020). It is known that activating astrocytic DRD2 can reduce astrocytic proliferation and inhibit the activation of astrocyte (Tanaka et al., 2011; Dominguez-Meijide et al., 2017; Du et al., 2018). In addition to DRD2, DRD1 can modulate astrogliosis to promote M2-like phenotype of astrocyte (Xia et al., 2019). One study showed that DRD1 activation increased excitability and evoked firing of the BLA neurons, whereas DRD2 activation increased input resistance, which indicated that dopamine system in the amygdala are implicated in the modulation of electrophysiological features of the amygdala neurons (Kroner et al., 2005). Based on the above results, we speculated EA treatment might upregulate DA synthesis and release and increase the expression of DRD1 and DRD2 in the amygdala, which subsequently activates the DRD1 and DRD2 signals, then suppresses astrocyte activation and inhibits excessive neuroinflammation, and finally results in alleviating pain and improving negative emotion.

Several limitations of this study should be considered. First, it should be clear in which cell types in the amygdala the up-regulated DRD1/DRD2 by EA treatment is expressed, and it should also be assessed what are the effects of EA on the activity of neurons and glial cells of the amygdala. Further studies are also needed to elucidate the contribution of the dopamine system in the amygdala to the effects of EA by using pharmaceutical antagonism or DRD1/DRD2 knockout animals. Second, the changes in the activity of TH should better be detected. Third, how EA regulates the dopamine system in the amygdala and what is the exact role of dopamine system in astrocyte activation inhibition by EA were not clarified.

In conclusion, the present results provide direct evidence that EA alleviates mechanical allodynia and the emotional dysfunction induced by NeuP. The regulation of the DA system in the amygdala may be involved in the effects of EA, which may underlie the mechanism of the therapeutic effect of EA in NeuP.

## Data Availability Statement

The original contributions presented in the study are included in the article/supplementary material, further inquiries can be directed to the corresponding author/s.

## Ethics Statement

The animal study was reviewed and approved by the Institutional Animal Care Committee of Yueyang Hospital of Integrated Traditional Chinese and Western Medicine, the Shanghai University of Traditional Chinese Medicine, Shanghai, China (YYLAC-2019-047).

## Author Contributions

KW, YY, and JZ conceived and designed the experiments. X-HZ, C-CF, and L-JP performed the experiments and analyzed the data. Y-NZ, LC, and X-QW helped with the behavior test experiments and analyzing the data. KW, YY, X-HZ, and JZ wrote and modified the manuscript. All authors discussed the results and reviewed the manuscript.

## Conflict of Interest

The authors declare that the research was conducted in the absence of any commercial or financial relationships that could be construed as a potential conflict of interest.

## References

[B1] Alles S. R. A. Smith P. A. (2018). Etiology and pharmacology of neuropathic pain. *Pharmacol. Rev.* 70 315–347. 10.1124/pr.117.014399 29500312

[B2] Arimura D. Shinohara K. Takahashi Y. Sugimura Y. K. Sugimoto M. Tsurugizawa T. (2019). Primary role of the amygdala in spontaneous inflammatory pain- associated activation of pain networks - a chemogenetic manganese-enhanced MRI approach. *Front. Neural Circ.* 13:58. 10.3389/fncir.2019.00058 PMC6779784 31632244

[B3] Bai Y. Ouyang S. L. Bai Y. J. Wu D. H. (2017). Treatment for persistent somatoform pain disorder via electroacupuncture and a low dosage of fluoxetine hydrochloride. *Integr. Med.* 16 28–31. PMC6415627 30881254

[B4] Barcelon E. E. Cho W. H. Jun S. B. Lee S. J. (2019). Brain microglial activation in chronic pain-associated affective disorder. *Front. Neurosci.* 13:213. 10.3389/fnins.2019.00213 PMC6436078 30949019

[B5] Baron R. (2006). Mechanisms of disease: neuropathic pain–a clinical perspective. *Nat. Clin. Pract. Neurol.* 2 95–106. 10.1038/ncpneuro0113 16932531

[B6] Bergman O. Ahs F. Furmark T. Appel L. Linnman C. Faria V. (2014). Association between amygdala reactivity and a dopamine transporter gene polymorphism. *Transl. Psychiatry* 4:e420. 10.1038/tp.2014.50 25093598 PMC4150236

[B7] Burke N. N. Geoghegan E. Kerr D. M. Moriarty O. Finn D. P. Roche M. (2013). Altered neuropathic pain behaviour in a rat model of depression is associated with changes in inflammatory gene expression in the amygdala. *Genes Brain Behav.* 12 705–713. 23957449 10.1111/gbb.12080

[B8] Chen J. Song Y. Yang J. Zhang Y. Zhao P. Zhu X. J. (2013). The contribution of TNF-alpha in the amygdala to anxiety in mice with persistent inflammatory pain. *Neurosci. Lett.* 541 275–280. 10.1016/j.neulet.2013.02.005 23415758

[B9] Clarkson B. D. S. Kahoud R. J. McCarthy C. B. Howe C. L. (2017). Inflammatory cytokine-induced changes in neural network activity measured by waveform analysis of high-content calcium imaging in murine cortical neurons. *Sci. Rep.* 7:9037. 10.1038/s41598-017-09182-5 PMC5567248 28831096

[B10] Colonna M. Butovsky O. (2017). Microglia function in the central nervous system during health and neurodegeneration. *Annu. Rev. Immunol.* 35 441–468. 10.1146/annurev-immunol-051116-052358 28226226 PMC8167938

[B11] Corder G. Ahanonu B. Grewe B. F. Wang D. Schnitzer M. J. Scherrer G. (2019). An amygdalar neural ensemble that encodes the unpleasantness of pain. *Science* 363 276–281. 10.1126/science.aap8586 30655440 PMC6450685

[B12] Descalzi G. Mitsi V. Purushothaman I. Gaspari S. Avrampou K. Loh Y. E. (2017). Neuropathic pain promotes adaptive changes in gene expression in brain networks involved in stress and depression. *Sci. Signal.* 10:eaaj1549. 10.1126/scisignal.aaj1549 28325815 PMC5524975

[B13] Dominguez-Meijide A. Rodriguez-Perez A. I. Diaz-Ruiz C. Guerra M. J. Labandeira-Garcia J. L. (2017). Dopamine modulates astroglial and microglial activity via glial renin-angiotensin system in cultures. *Brain Behav. Immun.* 62 277–290. 10.1016/j.bbi.2017.02.013 28232171

[B14] Du R. H. Zhou Y. Xia M. L. Lu M. Ding J. H. Hu G. (2018). alpha-Synuclein disrupts the anti-inflammatory role of Drd2 via interfering beta-arrestin2-TAB1 interaction in astrocytes. *J. Neuroinflammation* 15:258. 10.1186/s12974-018-1302-6 PMC6131810 30200997

[B15] Fang J. Jin Z. Wang Y. Li K. Kong J. Nixon E. E. (2009). The salient characteristics of the central effects of acupuncture needling: limbic-paralimbic-neocortical network modulation. *Hum. Brain Mapp.* 30 1196–1206. 10.1002/hbm.20583 18571795 PMC6871074

[B16] Finnerup N. B. Attal N. Haroutounian S. McNicol E. Baron R. Dworkin R. H. (2015). Pharmacotherapy for neuropathic pain in adults: a systematic review and meta-analysis. *Lancet Neurol.* 14 162–173. 25575710 10.1016/S1474-4422(14)70251-0 PMC4493167

[B17] Guan S. Y. Zhang K. Wang X. S. Yang L. Feng B. Tian D. D. (2020). Anxiolytic effects of polydatin through the blockade of neuroinflammation in a chronic pain mouse model. *Mol. Pain* 16:1744806919900717. 10.1177/1744806919900717 PMC6977205 31964240

[B18] Gui W. S. Wei X. Mai C. L. Murugan M. Wu L. J. Xin W. J. (2016). Interleukin-1beta overproduction is a common cause for neuropathic pain, memory deficit, and depression following peripheral nerve injury in rodents. *Mol. Pain* 12:1744806916646784. 10.1177/1744806916646784 PMC4956151 27175012

[B19] Guo X. Zhao Y. Huang F. Li S. Luo M. Wang Y. (2020). Effects of transcutaneous auricular vagus nerve stimulation on peripheral and central tumor necrosis factor alpha in rats with depression-chronic somatic pain comorbidity. *Neural Plast.* 2020:8885729. 10.1155/2020/8885729 PMC7599410 33144854

[B20] He L. Xu R. Chen Y. Liu X. Pan Y. Cao S. (2019). Intra-CA1 administration of minocycline alters the expression of inflammation-related genes in hippocampus of CCI Rats. *Front. Mol. Neurosci.* 12:248. 10.3389/fnmol.2019.00248 PMC6822549 31708740

[B21] Hiroi N. Brown J. R. Haile C. N. Ye H. Greenberg M. E. Nestler E. J. (1997). FosB mutant mice: loss of chronic cocaine induction of Fos-related proteins and heightened sensitivity to cocaine’s psychomotor and rewarding effects. *Proc. Natl. Acad. Sci. U.S.A.* 94 10397–10402. 10.1073/pnas.94.19.10397 9294222 PMC23374

[B22] Janak P. H. Tye K. M. (2015). From circuits to behaviour in the amygdala. *Nature* 517 284–292. 10.1038/nature14188 25592533 PMC4565157

[B23] Jang J. H. Song E. M. Do Y. H. Ahn S. Oh J. Y. Hwang T. Y. (2020). Acupuncture alleviates chronic pain and comorbid conditions in a mouse model of neuropathic pain: the involvement of DNA methylation in the prefrontal cortex. *Pain* 162 514–530. 10.1097/j.pain.0000000000002031 32796318 PMC7808350

[B24] Jha M. K. Jo M. Kim J. H. Suk K. (2019). Microglia-astrocyte crosstalk: an intimate molecular conversation. *Neuroscientist* 25 227–240. 10.1177/1073858418783959 29931997

[B25] Ji G. Neugebauer V. (2020). Kappa opioid receptors in the central amygdala modulate spinal nociceptive processing through an action on amygdala CRF neurons. *Mol. Brain* 13:128. 10.1186/s13041-020-00669-3 PMC7501648 32948219

[B26] Ji R. R. Xu Z. Z. Gao Y. J. (2014). Emerging targets in neuroinflammation-driven chronic pain. *Nat. Rev. Drug Discov.* 13 533–548. 10.1038/nrd4334 24948120 PMC4228377

[B27] Ju Z. Cui H. Guo X. Yang H. He J. Wang K. (2013). Molecular mechanisms underlying the effects of acupuncture on neuropathic pain. *Neural Regen. Res.* 8 2350–2359. 25206545 10.3969/j.issn.1673-5374.2013.25.006 PMC4146043

[B28] Khan W. U. Michelini G. Battaglia M. (2020). Twin studies of the covariation of pain with depression and anxiety: a systematic review and re-evaluation of critical needs. *Neurosci. Biobehav. Rev.* 111 135–148. 10.1016/j.neubiorev.2020.01.015 31954722

[B29] Kim B. Yoon S. Nakajima R. Lee H. J. Lim H. J. Lee Y. K. (2018). Dopamine D2 receptor-mediated circuit from the central amygdala to the bed nucleus of the stria terminalis regulates impulsive behavior. *Proc. Natl. Acad. Sci. U.S.A.* 115 E10730–E10739. 30348762 10.1073/pnas.1811664115 PMC6233075

[B30] Kirtley O. J. Rodham K. Crane C. (2020). Understanding suicidal ideation and behaviour in individuals with chronic pain: a review of the role of novel transdiagnostic psychological factors. *Lancet Psychiatry* 7 282–290. 10.1016/s2215-0366(19)30288-3 31982031

[B31] Klaus F. Paterna J. C. Marzorati E. Sigrist H. Gotze L. Schwendener S. (2016). Differential effects of peripheral and brain tumor necrosis factor on inflammation, sickness, emotional behavior and memory in mice. *Brain Behav. Immun.* 58 310–326. 10.1016/j.bbi.2016.08.001 27515532

[B32] Kroner S. Rosenkranz J. A. Grace A. A. Barrionuevo G. (2005). Dopamine modulates excitability of basolateral amygdala neurons in vitro. *J. Neurophysiol.* 93 1598–1610. 10.1152/jn.00843.2004 15537813

[B33] Lee J. H. Lee S. Kim J. H. (2017). Amygdala circuits for fear memory: a key role for dopamine regulation. *Neuroscientist* 23 542–553. 10.1177/1073858416679936 27872341

[B34] Li Q. Yue N. Liu S. B. Wang Z. F. Mi W. L. Jiang J. W. (2014). Effects of chronic electroacupuncture on depression- and anxiety-like behaviors in rats with chronic neuropathic pain. *Evid. Based Complement. Alternat. Med.* 2014:158987. 10.1155/2014/158987 PMC3984799 24795763

[B35] Liang Y. Qiu Y. Du J. Liu J. Fang J. Zhu J. (2016). Inhibition of spinal microglia and astrocytes contributes to the anti-allodynic effect of electroacupuncture in neuropathic pain induced by spinal nerve ligation. *Acupunct. Med.* 34 40–47. 10.1136/acupmed-2015-010773 26177687

[B36] Lin L. Yu L. Xiang H. Hu X. Yuan X. Zhu H. (2019). Effects of acupuncture on behavioral stereotypies and brain dopamine system in mice as a model of tourette syndrome. *Front. Behav. Neurosci.* 13:239. 10.3389/fnbeh.2019.00239 PMC6803462 31680895

[B37] Linnerbauer M. Wheeler M. A. Quintana F. J. (2020). Astrocyte crosstalk in CNS inflammation. *Neuron* 108 608–622. 10.1016/j.neuron.2020.08.012 32898475 PMC7704785

[B38] Liu S. Wang Z. F. Su Y. S. Ray R. S. Jing X. H. Wang Y. Q. (2020). Somatotopic organization and intensity dependence in driving distinct NPY-expressing sympathetic pathways by electroacupuncture. *Neuron* 108 436–450 e437. 32791039 10.1016/j.neuron.2020.07.015 PMC7666081

[B39] Maletic V. Raison C. L. (2009). Neurobiology of depression, fibromyalgia and neuropathic pain. *Front. Biosci.* 14 5291–5338. 10.2741/3598 19482616

[B40] Miller S. M. Marcotulli D. Shen A. Zweifel L. S. (2019). Divergent medial amygdala projections regulate approach-avoidance conflict behavior. *Nat. Neurosci.* 22 565–575. 10.1038/s41593-019-0337-z 30804529 PMC6446555

[B41] Ming Z. Criswell H. E. Breese G. R. (2013). Evidence for TNFalpha action on excitatory and inhibitory neurotransmission in the central amygdala: a brain site influenced by stress. *Brain Behav. Immun.* 33 102–111. 10.1016/j.bbi.2013.06.001 23770090 PMC3775850

[B42] Mitsi V. Terzi D. Purushothaman I. Manouras L. Gaspari S. Neve R. L. (2015). RGS9-2–controlled adaptations in the striatum determine the onset of action and efficacy of antidepressants in neuropathic pain states. *Proc. Natl. Acad. Sci. U.S.A.* 112 E5088–E5097. 26305935 10.1073/pnas.1504283112 PMC4568688

[B43] Neugebauer V. (2015). Amygdala pain mechanisms. *Handb. Exp. Pharmacol.* 227 261–284. 10.1007/978-3-662-46450-2_13 25846623 PMC4701385

[B44] Pavlova I. V. Rysakova M. P. Spivak J. S. Broshevitskaya N. D. Aksenova J. V. Salozhin S. V. (2020). Effects of decreases in dopamine (D1 and D2) receptor expression in the basolateral amygdala of rats on conditioned defensive reflexes. *Neurosci. Behav. Phys.* 50 315–326. 10.1007/s11055-020-00903-4

[B45] Prossin A. R. Zalcman S. S. Heitzeg M. M. Koch A. E. Campbell P. L. Phan K. L. (2015). Dynamic interactions between plasma IL-1 family cytokines and central endogenous opioid neurotransmitter function in humans. *Neuropsychopharmacology* 40 554–565. 10.1038/npp.2014.202 25139063 PMC4289943

[B46] Prut L. Belzung C. (2003). The open field as a paradigm to measure the effects of drugs on anxiety-like behaviors: a review. *Eur. J. Pharmacol.* 463 3–33. 10.1016/s0014-2999(03)01272-x 12600700

[B47] Qiu J. Yan Z. Tao K. Li Y. Li J. Dong Y. (2016). Sinomenine activates astrocytic dopamine D2 receptors and alleviates neuroinflammatory injury via the CRYAB/STAT3 pathway after ischemic stroke in mice. *J. Neuroinflammation* 13:263. 10.1186/s12974-016-0739-8 PMC5057372 27724964

[B48] Rouwette T. Vanelderen P. Roubos E. W. Kozicz T. Vissers K. (2012). The amygdala, a relay station for switching on and off pain. *Eur. J. Pain* 16 782–792. 10.1002/j.1532-2149.2011.00071.x 22337528

[B49] Shao W. Zhang S. Z. Tang M. Zhang X. H. Zhou Z. Yin Y. Q. (2013). Suppression of neuroinflammation by astrocytic dopamine D2 receptors via alphaB-crystallin. *Nature* 494 90–94. 10.1038/nature11748 23242137

[B50] Tanaka K. Kanno T. Yanagisawa Y. Yasutake K. Hadano S. Yoshii F. (2011). Bromocriptine methylate suppresses glial inflammation and moderates disease progression in a mouse model of amyotrophic lateral sclerosis. *Exp. Neurol.* 232 41–52. 10.1016/j.expneurol.2011.08.001 21867702

[B51] Thompson J. M. Neugebauer V. (2017). Amygdala plasticity and pain. *Pain Res. Manag.* 2017:8296501. 10.1155/2017/8296501 PMC5742506 29302197

[B52] Torres-Rosas R. Yehia G. Pena G. Mishra P. del Rocio, Thompson-Bonilla M. (2014). Dopamine mediates vagal modulation of the immune system by electroacupuncture. *Nat. Med.* 20 291–295. 10.1038/nm.3479 24562381 PMC3949155

[B53] Toth C. Lander J. Wiebe S. (2009). The prevalence and impact of chronic pain with neuropathic pain symptoms in the general population. *Pain Med.* 10 918–929. 10.1111/j.1526-4637.2009.00655.x 19594844

[B54] Vickers A. J. Vertosick E. A. Lewith G. MacPherson H. Foster N. E. Sherman K. J. (2018). Acupuncture for chronic pain: update of an individual patient data meta-analysis. *J. Pain* 19 455–474. 10.1016/j.jpain.2017.11.005 29198932 PMC5927830

[B55] Walker A. K. Kavelaars A. Heijnen C. J. Dantzer R. (2014). Neuroinflammation and comorbidity of pain and depression. *Pharmacol. Rev.* 66 80–101. 10.1124/pr.113.008144 24335193 PMC3880465

[B56] Wang K. Zeng L. Zhou Y. R. Zhou Y. F. Zhao R. Yang L. P. (2018). [Electroacupuncture relieves pain by down-regulating expression of hippocampal high mobility group protein 1 and contents of TNF-alpha and IL-1 beta in rats with chronic neuropathic pain]. *Zhen Ci Yan Jiu* 43 480–484. 30232849 10.13702/j.1000-0607.170929

[B57] Wang X. Wang Z. Liu J. Chen J. Liu X. Nie G. (2016). Repeated acupuncture treatments modulate amygdala resting state functional connectivity of depressive patients. *Neuroimage Clin.* 12 746–752. 10.1016/j.nicl.2016.07.011 27812501 PMC5079358

[B58] Xia Q. P. Cheng Z. Y. He L. (2019). The modulatory role of dopamine receptors in brain neuroinflammation. *Int. Immunopharmacol.* 76:105908. 10.1016/j.intimp.2019.105908 31622861

[B59] Xu Z. Fang J. Xiang X. Sun H. Wang S. Du J. (2020). Electroacupuncture alleviates pain-related emotion by upregulating the expression of NPS and its receptor npsr in the anterior cingulate cortex and hypothalamus. *Evid. Based Complement Alternat. Med.* 2020: 8630368. 10.1155/2020/8630368 PMC7035524 32104195

[B60] Yan B. Zhu S. Wang Y. Da G. Tian G. (2020). Effect of acupuncture on chronic pain with depression: a systematic review. *Evid. Based Complement Alternat. Med.* 2020:7479459. 10.1155/2020/7479459 PMC7334776 32714417

[B61] Yan Y. Jiang W. Liu L. Wang X. Ding C. Tian Z. (2015). Dopamine controls systemic inflammation through inhibition of NLRP3 inflammasome. *Cell* 160 62–73. 10.1016/j.cell.2014.11.047 25594175

[B62] Yang S. Boudier-Reveret M. Choo Y. J. Chang M. C. (2020). Association between chronic pain and alterations in the mesolimbic dopaminergic system. *Brain Sci.* 10:701. 10.3390/brainsci10100701 33023226 PMC7600461

[B63] Ye Y. Li H. Yang J. W. Wang X. R. Shi G. X. Yan C. Q. (2017). Acupuncture attenuated vascular dementia-induced hippocampal long-term potentiation impairments via activation of D1/D5 receptors. *Stroke* 48 1044–1051. 10.1161/strokeaha.116.014696 28289242

[B64] Zhang R. Lao L. Ren K. Berman B. M. (2014). Mechanisms of acupuncture-electroacupuncture on persistent pain. *Anesthesiology* 120 482–503. 10.1097/aln.0000000000000101 24322588 PMC3947586

[B65] Zhao Z. Q. (2008). Neural mechanism underlying acupuncture analgesia. *Prog. Neurobiol.* 85 355–375. 10.1016/j.pneurobio.2008.05.004 18582529

